# Contribution of white matter microstructure to diffusion tensor image analysis along perivascular space in obstructive sleep apnea

**DOI:** 10.1007/s11604-025-01838-x

**Published:** 2025-07-24

**Authors:** Toshiaki Taoka, Kunihiro Iwamoto, Seiko Miyata, Rintaro Ito, Koji Kamagata, Rei Nakamichi, Toshiki Nakane, Mami Iima, Hiroshige Fujishiro, Masashi Ikeda, Kazushige Ichikawa, Akifumi Kamiunten, Nobuyasu Ichinose, Junko Kikuta, Shigeki Aoki, Shinji Naganawa

**Affiliations:** 1 Department of Innovative Biomedical Visualization (iBMV), Nagoya University, 65 Tsurumai-cho, Showa-ku, Nagoya, Aichi 466-8550 Japan; 2 Department of Radiology, Nagoya University, Nagoya, Japan; 3 Department of Psychiatry, Nagoya University, Nagoya, Japan; 4 Department of Radiology, Juntendo University of Medicine, Tokyo, Japan; 5 Department of Fundamental Development for Advanced Low Invasive Diagnostic Imaging, Nagoya University, Nagoya, Japan; 6 Canon Medical Systems Corporation, Otawara, Japan

**Keywords:** Glymphatic system, DTI-ALPS, White matter microstructure, Obstructive sleep apnea, Polysomnography

## Abstract

**Purpose:**

We aimed to evaluate whether the ALPS index derived from diffusion tensor image analysis along the perivascular space (DTI-ALPS) is influenced by white matter fibres within the analysis region, particularly commissural fibres from the corpus callosum that traverse this area in psychiatric patients with suspected obstructive sleep apnea (OSA). We also investigated associations between diffusion-based parameters, sleep-related data, and neurofluid-related imaging metrics.

**Methods:**

Fifty participants with OSA underwent brain magnetic resonance imaging and polysomnography, including diffusion tensor and structural sequences. Among them, 8 participants had no psychiatric comorbidities, while the remaining 42 had various psychiatric disorders in addition to OSA. Diffusion-based parameters were obtained, and both the original and variant ALPS index were calculated. Correlation analyses were conducted with sleep-related data and neurofluid-related imaging parameters, including choroid plexus volume (CPV) and volume of white matter lesion burden (WMHV). Mediation analyses were also performed to explore the influence of white matter diffusivity on the perivascular diffusivity index.

**Results:**

The ALPS index showed weak to moderate correlations with multiple sleep-related variables. It also correlated with CPV and WMHV. Mediation analyses demonstrated that diffusivity within white matter fibres was associated with the ALPS index. Moreover, variant ALPS indices measured in the corpus callosum may reflect fluid motion in the direction of perivascular spaces.

**Conclusion:**

These findings suggest that the ALPS index is influenced by both diffusivity along perivascular spaces and white matter microstructure, particularly commissural fibres. Although it should not be regarded as a highly specific marker of perivascular space function, variant indices support partial perivascular contribution. Furthermore, associations with sleep and neurofluid-related metrics imply that white matter architecture and inter-fibre spaces may serve as plausible routes for interstitial fluid flow.

**Supplementary Information:**

The online version contains supplementary material available at 10.1007/s11604-025-01838-x.

## Introduction

The glymphatic system hypothesis, proposed more than a decade ago, suggests that the cerebrospinal and interstitial fluid dynamics play key roles in brain waste clearance [1]. Since this report, there has been a growing interest in fluid dynamics related to the brain, and the evaluation of fluid dynamics related to the nervous system as “neurofluid dynamics” has been promoted [2–5]. This movement has led to the development of noninvasive techniques for evaluating neurofluid dynamics [2, 6, 7]. One such method, diffusion tensor image analysis along the perivascular space (DTI-ALPS), introduced in 2017, estimates interstitial fluid dynamics using the ALPS index [8]. Subsequent studies have shown correlations between ALPS index values and various physiological and pathological conditions [9–18].

The efficiency of the glymphatic system increases during sleep [19]. Obstructive sleep apnea (OSA) is one of the most common sleep disorders, affecting approximately 9–38% of adults globally [20], and there have been several reports of this disease using DTI-ALPS [21–23]. Patients with severe OSA have higher perivascular space volume fractions, increased extracellular free water, and lower ALPS index values [23]. However, the ALPS index only reflects the characteristics of diffusion in the perivascular space and does not directly show the movement of the interstitial fluid. Therefore, further verification and technological development are necessary to establish an ALPS method to evaluate the function of the glymphatic system [24].

DTI-ALPS also faces other methodological criticisms and is limited to a specific region of interest (ALPS-ROI) in the projection and association of fiber areas adjacent to the lateral ventricle. Glymphatic function throughout the rest of the brain is currently unknown [25]. Furthermore, the ALPS index may be influenced by crossing fibers, particularly commissural fibers traversing the corpus callosum, as DTI assumes a single dominant fiber orientation per voxel and may not accurately reflect regions with complex fiber configurations [26–28]. To address these limitations, a multimodal imaging approach has been recommended [26]. Other methods under investigation include free water analysis, arterial spin labeling, choroid plexus volume (CPV), perivascular space volume, and white matter hypointensity volume (WMHV) measured on T1-weighted imaging, which represents white matter lesion burden [29–32].

In this context, we initiated the Multimodal Observation Of NeurofLuid through Imaging-based GlympHatic Transport (MOONLIGHT) study, designed to comprehensively evaluate sleep-related neurofluid dynamics using multimodal MRI techniques and polysomnography (PSG). To address potential confounding effects of complex fiber architecture, we introduced variant ALPS indices calculated in anatomically simpler regions of the corpus callosum, where fiber orientations are more uniform. The body of the corpus callosum, in particular, was selected for its anatomical continuity with the conventional ALPS-ROI. This study aimed to determine whether the ALPS index is affected by white matter fiber microstructure, specifically commissural fibers traversing the corpus callosum. We examined the correlations between diffusion-derived metrics—including both the conventional ALPS index and variant indices—and PSG-derived sleep parameters, choroid plexus volume (CPV), and white matter hypointensity volume (WMHV) measured on T1-weighted imaging, which represents white matter lesion burden. We further explored whether perivascular orientation influenced the variant ALPS indices.

## Materials and methods

### Participants

**Fig. 1:**
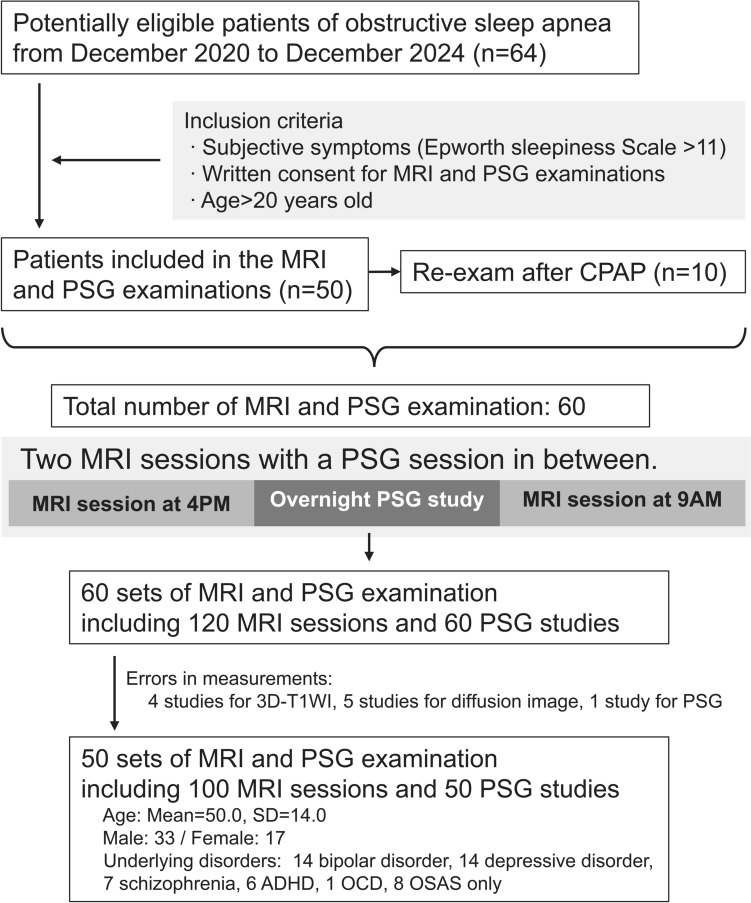
Study participant flow and procedure overview. This flowchart illustrates the recruitment pathway, inclusion/exclusion details, and the final dataset composition for the analyses

This prospective study was approved by the institutional review board (2020-0352) and registered in the University Hospital Medical Information Network Clinical Trials Registry (UMIN-CTR, R000045230). From December 2020 to December 2024, patients visiting the Department of Psychiatry with suspected OSA were included based on the following criteria: symptoms suggestive of OSA (e.g., snoring, obesity, sleepiness), agreement to undergo MRI and PSG, and age ≥ 20 years. In total, 64 patients were screened; 50 were eligible for the initial MRI and PSG, and 10 others agreed to retesting after initiating continuous positive airway pressure therapy, resulting in 60 MRI–PSG sets. Each consisted of MRI at 4 PM, overnight PSG (≥ 7 h), and MRI at 9 AM the next morning. After excluding 10 datasets due to incomplete data (T1-weighted images, 4; diffusion, 5; PSG, 1), 50 datasets remained (MRI, 100 sessions; PSG, 50). The final cohort (*n* = 50) had a mean age of 50.0 (SD 14.0) years, with 33 men and 17 women. All participants were diagnosed with obstructive sleep apnea (OSA) based on overnight PSG. Among them, 8 patients had no psychiatric comorbidities and were classified as OSA-only. The remaining 42 participants had comorbid psychiatric diagnoses, including bipolar disorder (*n* = 14), depression (*n* = 14), schizophrenia (*n* = 7), attention-deficit/hyperactivity disorder (ADHD; *n* = 6), and obsessive–compulsive disorder (OCD; *n* = 1). (Fig. 1). Among the final 50 participants, 9 underwent follow-up MRI after initiating CPAP therapy. As a result, the dataset includes both pre- and post-CPAP cases. However, the effect of CPAP was not modeled explicitly in this analysis due to sample size limitations.

### Overnight PSG

PSG data were analyzed to evaluate sleep architecture, respiratory function, and autonomic responses. All participants underwent standard PSG using Embla N7000 (Natus Neurology Incorporated) or PSG-1100 (Nihon Kohden Corporation). The results were evaluated according to the American Academy of Sleep Medicine Scoring Manual (version 2.5) for the night, between the evening and morning MRI examinations [33]. Sleep architecture was assessed based on the total sleep time, sleep latency, rapid eye movement (REM) sleep latency, wake after sleep onset (WASO), sleep efficiency, and proportion of time spent in each sleep stage, including Stage N1 (light sleep with reduced muscle tone), Stage N2 (intermediate sleep characterized by sleep spindles and K-complexes), Stage N3 (deep slow-wave sleep), and REM sleep. Respiratory parameters included the arousal index, apnea index, hypopnea index, apnea–hypopnea index, lowest recorded oxygen saturation, 3% oxygen desaturation index, and total duration of time with oxygen saturation below 90%.

### MRI acquisition

MR images were obtained using a 3 T clinical scanner (Vantage Centurian, Canon Medical Systems, Otawara). The first MRI session was performed at 4 PM before the PSG study (MRI/4PM). This was followed by overnight PSG. The second MRI session was performed at 9 AM the following morning after the PSG (MRI/9AM). Various images were obtained during the MRI session, including T1-weighted images and diffusion tensor images using echo-planar imaging. Diffusion tensor images was acquired by echo-planar imaging, repetition time = 6600 ms, echo time = 85 ms, diffusion time = 35.7 ms, motion-proving gradient = 12 axes, *b*-value = 1,000 s/mm^2^, anterior-to-posterior phase encode direction, FOV = 200 mm, matrix = 128 × 128 with interpolation to 256 × 256, slice thickness = 3 mm, slice number = 50, number of averaging = 2. We apply the top-up process to correct susceptibility-induced distortions; *b* = 0 images with posterior-to-anterior phase encoding were acquired. 3D T1-weighted MPRAGE scans were performed using the following parameters: repetition time = 6000 ms, echo time = 2.5 ms, inversion time = 800 ms, flip angle = 9°, bandwidth = 279 Hz, FOV = 250 mm, matrix = 256 × 256, slice thickness = 1.5 mm, slice number = 260.

### Image analysis

#### ALPS index

DTI data were processed using the FMRIB Software Library version 6.0 (FSL; Oxford Center for Functional MRI of the Brain, Oxford, UK). Preprocessing steps included eddy current correction using FSL’s “eddy” tool and susceptibility-induced distortion correction using the “topup” method with reversed phase-encoded b0 images. All diffusion datasets were visually inspected for motion artifacts, and five datasets showing substantial artifacts were excluded prior to analysis. Diffusivity maps were obtained for each participant along the x- (right left; Dxx), y- (anterior–posterior; Dyy), and z-axes (inferior-superior; Dzz). The fractional anisotropy map for all individuals was converted into the FMRIB58_FA standard space using linear and non-linear transformations.

The participant with the smallest degree of warping was selected for the ROI placement to ensure a uniform ROI setting across all cases. The ALPS-ROIs of the projection and association areas of the left and right hemispheres were set based on previous reports [30, 32, 34, 35]. The ALPS index was calculated as the ratio of the mean x-axis diffusivity in the projection area (projDxx) to the x-axis diffusivity in the association area (assocDxx), mean y-axis diffusivity in the projection area (projDyy), and z-axis diffusivity in the association area (assocDzz), as follows:

$${\text{ALPS index}} = {\text{mean }}\left( {{\text{projDxx}},{\text{ assocDxx}}} \right)/{\text{mean }}\left( {{\text{projDyy}},{\text{ assocDzz}}} \right)$$

ROI reproducibility was not assessed via inter-rater reliability because a common ALPS-ROI template was used for all participants. Specifically, a single subject with minimal non-linear warping during spatial normalization was selected, and ALPS-ROIs were defined in this subject. These ROIs were then applied to all participants in standard (FMRIB58_FA) space. This approach minimizes inter-individual variability in ROI placement. To ensure accurate registration, all non-linear warps were visually checked, and major distortions were excluded.

### ALPS indices in the corpus callosum

**Fig. 2:**
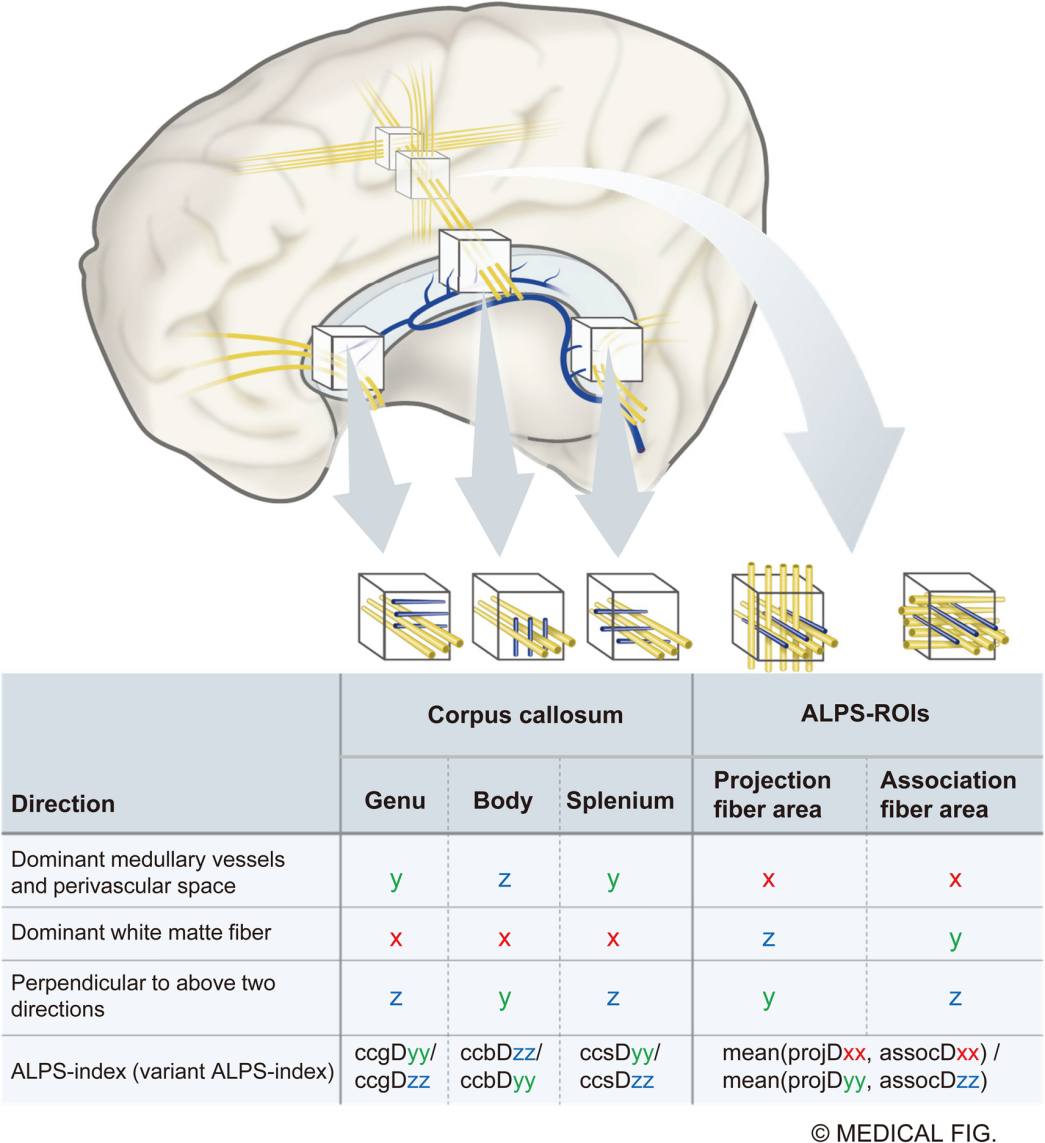
Definition of variant ALPS indices. Variant ALPS indices were set for the genu, body, and splenium of the corpus callosum. Whereas the white matter fibers of the corpus callosum run left-to-right along the x-axis, the orientation of the vessels differs depending on the region. In the genu of the corpus callosum, the vessels run primarily in the anteroposterior direction along the y-axis. In the corpus callosum, the vessels mainly ran vertically along the z-axis. In the splenium of the corpus callosum, the vessels run primarily in the anteroposterior direction along the y-axis. The figure also illustrates the ALPS-ROI (projection and association areas in the white matter outside the body of lateral ventricle). Each variant of the ALPS index is defined as the ratio of diffusivity along the vascular direction to that along the perpendicular direction. In the genus of the corpus callosum, ccgALPS was calculated as ccgDyy/ccgDzz. In the body of the corpus callosum, ccbALPS was calculated as ccbDzz/ccbDyy. In the splenium of the corpus callosum, the ccsALPS was calculated as ccsDyy/ccsDzz

Variant ALPS indices were set in three areas of the corpus callosum. Because the direction of the fibers and blood vessels in the corpus callosum is simple, it was thought that it would be possible to confirm the extent to which the direction of the perivascular space affects the diffusivity of the ROIs. The white matter fibers of the corpus callosum run from left-to-right or along the x-axis. In contrast, in the genu and splenium of the corpus callosum, the medullary arteries and veins run mainly in the anterior–posterior direction or the y-axis direction, while in the body of the corpus callosum, the medullary arteries and veins run mainly in the vertical direction or the z-axis direction [36, 37]. Based on these anatomical relationships, the following three variant ALPS indices related to the corpus callosum were calculated as the ratios of diffusivity along the direction of the medullary veins and arteries to that in the direction perpendicular to both the vascular orientation and the main white matter fiber orientation (Fig. 2). In the genu of the corpus callosum, ccgALPS was defined as ccgDyy/ccgDzz; in the body of the corpus callosum, ccbALPS as ccbDzz/ccbDyy; and in the splenium of the corpus callosum, ccsALPS was defined as ccsDyy/ccsDzz.

### Choroid plexus and white matter hypointensity volumes

For the volumetric analysis of the CPV and WMHV, 3D T1-weighted images were automatically segmented using FreeSurfer version 7.4.1 (https://surfer.nmr.mgh.harvard.edu/). The “recon-all” pipelines were used for brain segmentation. These steps included motion correction, normalization, automatic segmentation, and registration. Brain volume segmentation was based on an atlas containing probabilistic information regarding the location of the structures. Images were segmented by normalizing the participant data to a common space and applying a probabilistic atlas to image-specific voxel intensities to select the optimal segmentation [38], [39]. All FreeSurfer segmentations were visually inspected for quality assurance. Cases with obvious segmentation errors, such as ventricular overestimation or cortical mislabeling, were corrected manually using Freeview by modifying the brain mask or placing control points as appropriate. The total intracranial volume, CPV, and WMHV were determined. All automatic segmentations were quality-checked and approved by a neuroradiologist blinded to the sleep data. Relative CPV (rCPV) and relative WMHV (rWMHV) were assessed as ratios to the total intracranial volume, following previously reported methods [31, 32].

### Statistical analysis

#### Head motion quantification

Framewise displacement (FD) was calculated for each subject from the motion parameters estimated during eddy current correction (FSL eddy). For each subject, mean and maximum FD values were derived. To assess potential motion-related bias, Pearson’s correlation was calculated between mean FD and the ALPS index.

#### Correlations between imaging and PSG parameters

To examine how imaging parameters, including the ALPS index, correlated with PSG data, correlation analyses were conducted. For MRI sessions at 4 PM and 9 AM before and after overnight PSG, correlations were assessed between imaging-derived metrics (MRI/4PM and MRI/9AM) and sleep and respiratory measures from PSG. Imaging parameters included the ALPS index, variant indices (ccgALPS, ccbALPS, ccsALPS), other diffusion metrics, rCPV, and rWMHV. To assess temporal changes, differences in ALPS index and related diffusion parameters between the 4 PM and 9 AM MRI sessions (*Δ* values) were calculated. These Δ values were then analyzed in relation to PSG-derived sleep parameters. Descriptive statistics for ALPS index values at both time points and for ΔALPS (9 AM–4 PM) were summarized using the mean ± standard deviation and the median with interquartile range, and are reported in the main Results section. Pearson correlation was used, assuming approximate normality of imaging variables. Statistical significance was set at *p* < 0.05 (two-tailed), with Benjamini–Hochberg false discovery rate correction for multiple comparisons. Correlation strength was categorized as very weak (0–0.19), weak (0.20–0.39), moderate (0.40–0.59), strong (0.60–0.79), or very strong (0.80–1.00). To support transparency and reproducibility, p value heatmaps were also generated and are presented in the Supplementary Appendix (Fig. S1). These were intended to complement the *r*-value heatmaps presented in the main text by providing a statistical significance reference for each correlation.

#### Correlation analysis of diffusion parameters with rCPV and WHMV

##### Effect of ALPS index and variant ALPS indices on rCPV and rWHMV

To examine how the ALPS index and its variants correlate with glymphatic indicators, we performed correlation and multiple regression analyses using the full dataset, including MRI scans at 4 PM and 9 AM. Correlation coefficients and p values were calculated between the ALPS index, its variants, rCPV, and rWMHV, with FDR correction applied.

Multiple regression was then used to assess the relationship between diffusion indices and rCPV or rWMHV, adjusting for age and psychiatric conditions using 0/1 dummy variables. To account for potential confounding effects from psychiatric comorbidities and psychotropic medications, we included psychiatric diagnoses as covariates in the models. Although these factors may influence brain structure and function, this adjustment aimed to mitigate their impact within our statistical analyses. All continuous variables were standardized (mean = 0, SD = 1). Standardized regression coefficients and 95% confidence intervals were computed, and variance inflation factors were checked to rule out multicollinearity. Forest plots visualized these coefficients and their intervals. Statistical significance was defined as a 95% CI not crossing zero (≈ *p* < 0.05). Analyses were done separately for rCPV and rWMHV. To confirm the robustness of the findings, we additionally performed two sensitivity analyses: a subgroup analysis limited to OSA participants without psychiatric comorbidities (*n* = 8, 16 scans), and an analysis excluding the 9 participants who had received prior CPAP treatment. These complementary analyses allowed us to examine whether the main associations held true under more homogeneous clinical conditions.

##### Effects of diffusion parameters other than the ALPS index on rCPV and rWMHV

To investigate whether diffusivity metrics not used in the ALPS index formulation are also associated with glymphatic function, we performed additional multiple regression analyses using individual diffusion parameters obtained from the ALPS-ROI and corpus callosum ROI. These models included projection (projDyy, projDzz), association (assocDyy, assocDzz), and corpus callosum (ccbDxx, ccbDyy, ccbDzz) diffusivity values as predictors, adjusting for age and psychiatric status. As with the primary models, all variables were standardized and statistical significance was assessed based on the 95% confidence interval not crossing zero.

#### Evaluation of the effects of ALPS-ROI and corpus callosum diffusion parameters on the ALPS index

To assess whether diffusivity components orthogonal to the perivascular direction influence the ALPS index, mediation analyses were conducted using directional diffusivity measures within the ALPS-ROI and corpus callosum. In each model, the diffusivity aligned with the perivascular direction (e.g., assocDxx or projDxx) was set as the independent variable, and orthogonal diffusivity components (e.g., assocDyy, assocDzz, projDyy, projDzz) were tested as potential mediators. Similarly, to examine the influence of commissural fibers, mediation analyses were also performed using corpus callosum diffusivity measures (e.g., ccbDyy, ccbDzz) as mediators of the relationship between ALPS-ROI diffusivity and the ALPS index. All models were adjusted for age. Average causal mediation effects (ACME) were estimated using nonparametric bootstrapping, and statistical significance was defined by a 95% confidence interval not crossing zero. Both full and partial mediation patterns were considered.

The results of all 10 mediation models are summarized in Table 2, which presents the standardized coefficients (*β*) and 95% confidence intervals (CI) for the average causal mediation effect (ACME), average direct effect (ADE), and total effect derived from bootstrap simulations (1000 iterations). Mediation pathways are denoted using the format *X* (Independent Variable) → *M* (Mediator) → *Y* (Dependent Variable) to clarify the directionality of influence. The table is structured in alignment with Fig. 5, beginning with intra-regional mediation within the ALPS-ROI, followed by corpus callosum-related mediations, allowing for direct comparison between graphical and quantitative results.

#### Evaluation of the impact of the direction of medullary vessel on diffusivity

To assess whether the orientation of medullary vessels relative to white matter fibers affects diffusion characteristics, variant ALPS indices were calculated in three sub-regions of the corpus callosum: the genu, body, and splenium. These regions were selected because the fiber orientation is relatively homogeneous, and the course of medullary vessels in each region is anatomically distinct. Each variant index (ccgALPS, ccbALPS, and ccsALPS) was defined as the ratio of diffusivity along the presumed vascular direction to that along the orthogonal direction. One-sample *t* tests were planned to compare the mean values of each variant ALPS index against a reference value of 1.0, which represents isotropic diffusion.

## Results

### Head motion analysis

The mean framewise displacement (FD) across participants was 0.18 ± 0.07 mm. There was no significant correlation between mean FD and the ALPS index (*r* = 0.044, *p* = 0.652), suggesting that head motion did not substantially influence the ALPS index.

### Correlations between imaging and PSG parameters

Before assessing the correlations, descriptive statistics of the ALPS index were calculated. The mean ± standard deviation was 1.391 ± 0.261 at 4 PM and 1.419 ± 0.253 at 9 AM. The corresponding median values with interquartile ranges were 1.397 [1.253, 1.567] and 1.472 [1.300, 1.580], respectively. The difference between the two time points (ΔALPS) had a mean ± SD of 0.028 ± 0.199 and a median of 0.017 [–0.036, 0.077].

**Fig. 3:**
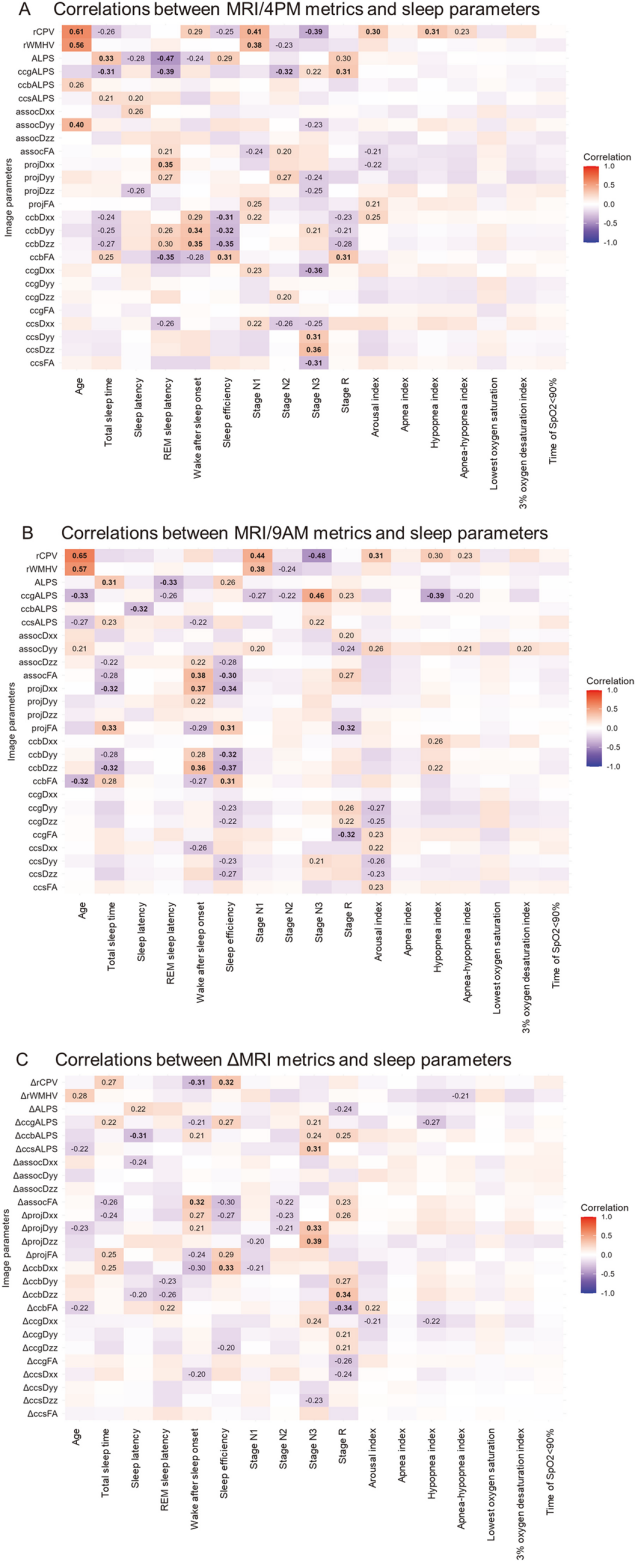
Imaging and sleep parameters and their associations. The imaging parameters included the rCPV, rWMHV, ALPS indices (ccgALPS, ccbALPS, and ccsALPS), and diffusion indices for each region (ALPS-ROI and corpus callosum). The PSG parameters included sleep stages, respiratory indices, and sleep assessment indices. Both correlation coefficients (*r*) and *p* values are visualized to reflect effect size and statistical significance, respectively. Note that discrepancies between the two may occur due to sample size or data distribution, and should be interpreted accordingly. **A** In MRI/4PM, the ALPS index showed an inverse correlation with REM sleep latency and a mild correlation with total sleep time and sleep efficiency. ccbALPS showed an inverse correlation with sleep latency, whereas white matter microstructure indices were related to sleep efficiency. The rCPV and rWMHV were associated with sleep metrics, age, and PLM indices. **B** In MRI/9AM, the ALPS index was correlated with the total sleep time and REM sleep latency. ccgALPS scores correlated with age and sleep stage. White matter microstructure indices, rCPV, and rWMHV, were associated with age and PLM indices. **C** Analysis of changes before and after sleep (*Δ* values) revealed significant correlations between ΔALPS index, variant indices, and sleep metrics such as sleep latency, sleep efficiency, WASO, and Stage N3. The rCPV and rWMHV are also related to various sleep metrics, PLM indices, and AHI

On MRI at 4PM (Fig. 3A), the perivascular diffusivity index showed a moderate inverse correlation with REM sleep latency (*r* = –0.47) and mild positive correlations with total sleep time (*r* = 0.33) and sleep efficiency (*r* = 0.29), with no association with age. Among the variant indices, ccbALPS showed a weak inverse correlation with sleep latency (*r* = –0.25). White matter diffusivity measures in the corpus callosum, including ccbDxx (*r* = 0.36), ccbDyy (*r* = 0.44), ccbDzz (*r* = 0.41), and ccbFA (*r* = 0.45), were positively correlated with sleep efficiency. rCPV was positively associated with age (*r* = 0.61), wake after sleep (*r* = 0.29), Stage N1 (*r* = 0.41), and arousal index (*r* = 0.30), while rWMHV showed positive correlations with age (*r* = 0.65) and Stage R (*r* = 0.38).

At 9AM (Fig. 3B), the index again correlated with total sleep time (*r* = 0.31) and REM sleep latency (*r* = –0.33), but not with age. ccgALPS showed inverse correlations with age (*r* = –0.33) and Stage N3 (*r* = –0.43). Sleep efficiency was correlated with ccbDyy (*r* = 0.30), ccbDzz (*r* = 0.35), and ccbFA (*r* = 0.31). rCPV and rWMHV were also associated with age and PLM-related metrics such as arousal index (*r* = 0.38) and time of SpO₂ < 90% (*r* = 0.31).

For sleep-related changes (*Δ*; Fig. 3C), ΔccsDzz showed an inverse correlation with Stage N3 (*r* = –0.41), and ΔrWMHV correlated with arousal index (*r* = 0.36) and hypopnea index (*r* = 0.36), suggesting associations between structural changes and respiratory instability.

A detailed summary of the corresponding p values is provided in Supplementary Fig. S1. As effect size and statistical reliability do not always align, some moderate r values did not reach conventional significance thresholds. This discrepancy underscores the complementary roles of correlation coefficients in reflecting association strength and p values in indicating statistical confidence.

### Correlation analysis of diffusion parameters with rCPV and WHMV

#### Effect of the ALPS index and variant ALPS index on rCPV and WHMV

**Fig. 4:**
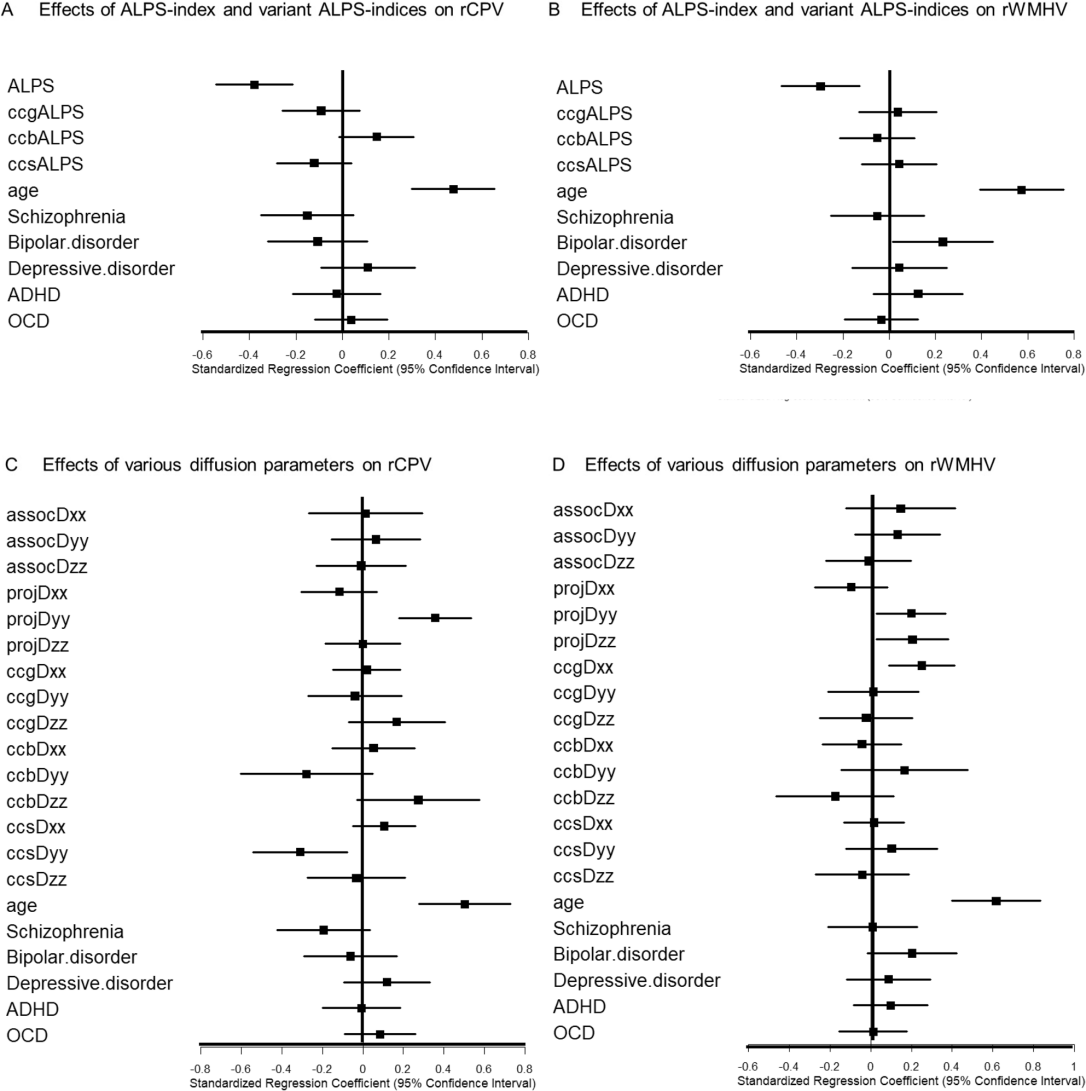
Regression analysis of factors affecting rCPV and Rwmhv. Evaluation of the effects of ALPS index, variant ALPS indices, and various diffusion parameters on rCPV and rWMHV, shown through forest plots of standardized regression coefficients (with 95% confidence intervals) of the effects of each parameter on the rCPV and rWMHV. All analyses were performed using multiple regression analysis adjusted for age and underlying psychiatric disorders as covariates. **A** The effects of the ALPS index and variant ALPS indices on rCPV age show a clear influence. The ALPS index shows a significant correlation with rCPV. In addition, ccgALPS, located in the genu of the corpus callosum, shows a significant correlation. No significant effects were observed on psychiatric disorders. **B** Effects of the ALPS index and variant ALPS indices on rWMHV. The ALPS index shows a significant correlation with rWMHV. In addition, ccgALPS shows a significant correlation. Age also has a significant effect. No significant effects are observed on psychiatric disorders. **C** Effects of various diffusion parameters on rCPV standardized analysis adjusted for age and psychiatric disorders showed that projDyy had a significant effect on rCPV. Age was also confirmed to be a significant influencing factor; however, no significant effects were observed for psychiatric disorders. **D** Effects of various diffusion parameters on rWMHV. In the standardized analysis adjusted for age and psychiatric disorders, projDyy, projDzz, and ccgDxx had significant effects on rWMHV. Age was also confirmed to be a significant influencing factor; however, no significant effects were observed for psychiatric disorders

Significant correlations were observed between the ALPS index and both rCPV and rWMHV, which reflect distinct aspects of glymphatic function. Likewise, ccgALPS, derived from the genu of the corpus callosum, was significantly associated with both rCPV and rWMHV (Table 1). In the analysis adjusted for age and background diseases using standardized variables (Fig. 4A, B), age remained a significant factor for both rCPV and rWMHV, while the ALPS index also retained a significant effect. Although bipolar disorder showed a slight association in some models, its influence was small and unlikely to affect the overall interpretation. Variant ALPS indices other than ccgALPS did not show any significant effects. To confirm the robustness of these associations, we conducted two sensitivity analyses. In a subgroup of OSA participants without psychiatric comorbidities (*n* = 8, 16 scans), the ALPS index remained significantly and negatively associated with both rCPV (*β* = − 0.00075, 95% CI [− 0.00147, − 0.00004], *p* = 0.041) and rWMHV (*β* = − 0.00102, 95% CI [− 0.00196, − 0.00009], *p* = 0.035). After excluding the 9 participants who had received CPAP treatment, the associations remained significant for both rCPV (*β* = − 0.00053, 95% CI [− 0.00079, − 0.00027], *p* = 0.0001) and rWMHV (*β* = − 0.00117, 95% CI [− 0.00174, − 0.00060], *p* = 0.0001).These findings demonstrate that the relationship between the ALPS index and glymphatic indicators was not driven by psychiatric status or CPAP history, reinforcing the robustness of the main results.

**Table 1:** Correlations between glymphatic imaging markers and ALPS indices

	rCPV	rWMHV	ALPS	ccgALPS	ccbALPS	ccsALPS
rCPV		**0.547435**	**−0.44001**	**−0.38065**	0.126145	−0.17691
rWMHV	**1.13E**−**08**		**−0.38406**	−0.28280	0.083471	−0.07244
ALPS	**9.10E**−**06**	**0.000133**		0.24106	0.03503	−0.09133
ccgALPS	**0.000154**	**0.005748**	**0.019257**		−0.02314	−0.08105
ccbALPS	0.225702	0.423799	0.737486	0.824813		0.00302
ccsALPS	0.088062	0.487766	0.381328	0.437384	0.976987	

Correlation matrix showing Pearson’s correlation coefficients (upper triangle) and associated p values (lower triangle) between the ALPS index, its regional variants (ccgALPS: corpus callosum–genu, ccbALPS: corpus callosum–body, ccsALPS: corpus callosum–splenium), relative choroid plexus volume (rCPV), and relative white matter hyperintensity volume (rWMHV). Significant negative correlations were observed between rCPV and both the ALPS index and ccgALPS, as well as between rWMHV and the ALPS index and ccgALPS. No significant correlations were found for ccbALPS or ccsALPS. Statistically significant correlations (*p* < 0.05) are highlighted in bold in the main text

#### Effects of diffusion parameters other than the ALPS index on rCPV and rWMHV

In the analysis where the effects of the diffusion indices other than the ALPS index on rCPV and rWMHV were adjusted for age and underlying disease and the variables were standardized, the effect of age was also significant (Fig. 4C, D). ProjDyy showed a significant effect on rCPV. In the rWMHV analysis, projDyy, projDzz, and ccgDxx exhibited significant effects.

### Effects of ALPS-ROI and corpus callosum diffusion parameters on the ALPS index

**Fig. 5:**
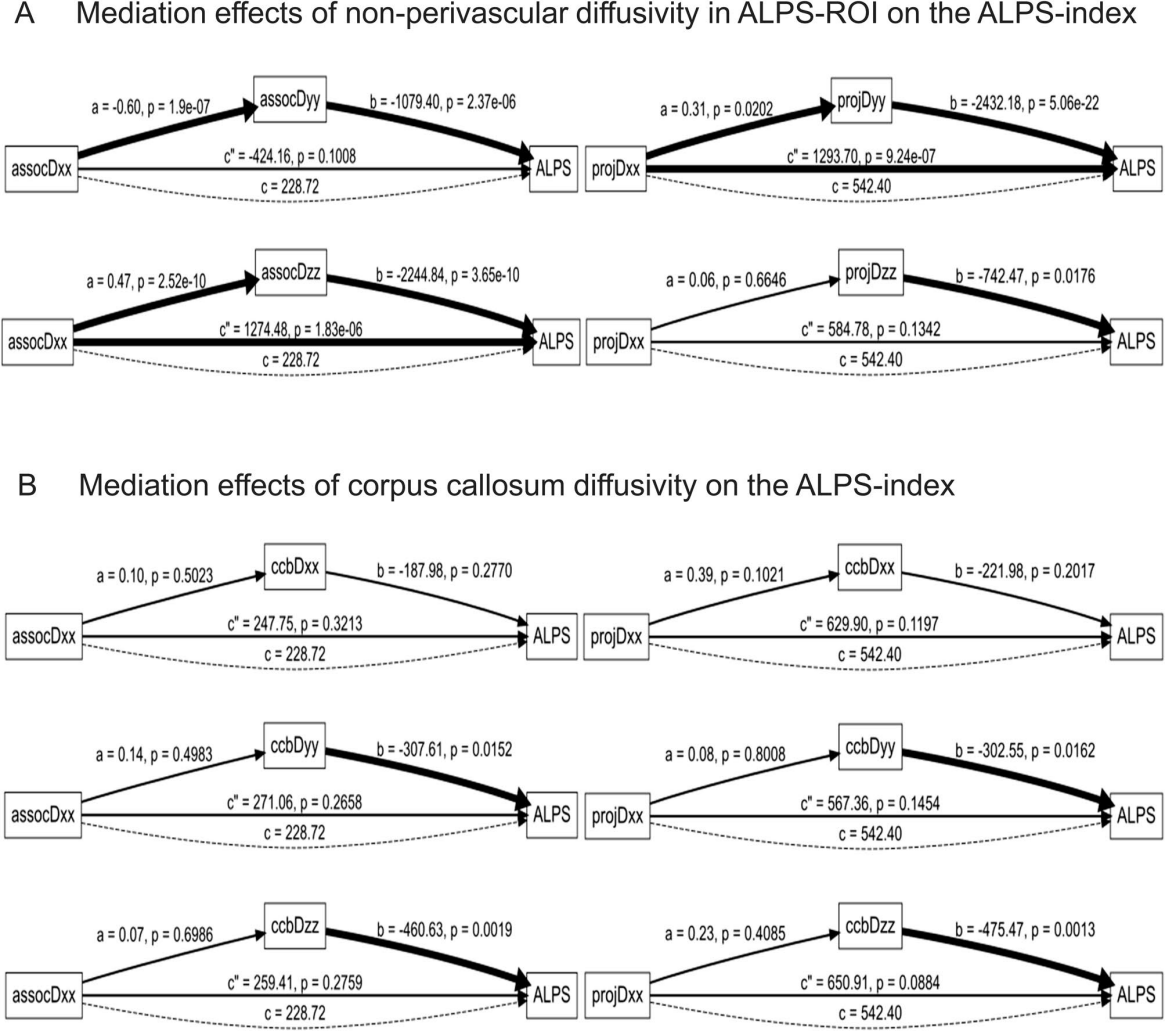
Mediation analysis of diffusion parameters and ALPS index. The effects of the diffusion parameters in the ALPS-ROI and corpus callosum on the ALPS index using mediation analyses. **A** In the ALPS-ROI, mediation analyses showed that diffusivity orthogonal to the perivascular space—particularly assocDyy and projDyy—significantly mediated the relationship between perivascular diffusivity (Dxx) and the ALPS index, as reflected by statistically significant Average Causal Mediation Effects. **B** In the corpus callosum, ccbDyy significantly mediated the association between assocDxx and the ALPS index. Although ccbDzz did not show a significant mediation effect, its association with the ALPS index via the mediator-to-outcome path (b-path) was statistically significant, suggesting that both commissural diffusion directions may independently contribute to the ALPS index

To explore whether directional diffusivity orthogonal to the perivascular space affects the ALPS index, we conducted mediation analyses using ALPS-ROI diffusivity components (assocDyy and assocDzz) as mediators of the relationship between perivascular-oriented diffusivity (assocDxx) and the ALPS index (Fig. 5A). Notably, when assocDyy was used as the mediator, both the a-path (assocDxx → assocDyy, *p* < 0.001) and the b-path (assocDyy → ALPS, *p* < 0.001) were statistically significant, and the average causal mediation effect (ACME) was also significant (*p* < 0.001), suggesting that assocDyy may play a potential mediating role in the association between assocDxx and the ALPS index. These results support the idea that diffusivity perpendicular to the perivascular direction contributes meaningfully to the ALPS index. Similarly, assocDzz showed significant a and b paths (both *p* < 0.001) and a significant negative mediation effect (ACME = −982.87, *p* < 0.001), even when adjusting for age.

In the projection fiber region, projDyy also demonstrated a significant mediation effect between projDxx and the ALPS index (ACME = −751.31, *p* = 0.002), whereas projDzz did not. These findings suggest that, in certain conditions, diffusion components not aligned with the perivascular direction may be more strongly associated with the ALPS index than those aligned.

In addition, we examined whether the ALPS index may be influenced by white matter microstructure in the corpus callosum through mediation analyses using corpus callosum diffusivity (ccbDxx, ccbDyy, and ccbDzz) as mediators (Fig. 5B). Among these models, only the one using ccbDyy as the mediator between assocDxx and the ALPS index yielded a statistically significant mediation effect. This model showed significant paths from assocDxx to ccbDyy (*p* < 0.001) and from ccbDyy to ALPS (*p* < 0.001), with a significant indirect effect (ACME = −364.75, *p* < 0.001), corresponding to approximately 57% of the observed association. In contrast, other combinations involving ccbDxx or ccbDzz, or those using projDxx as the independent variable, did not show significant mediation.

While significant mediation effects require both the predictor-to-mediator (a-path) and mediator-to-outcome (b-path) associations to be statistically supported, it is worth noting that a significant effect of the mediator on the outcome (b-path) alone may still indicate that the mediator is a critical determinant of the dependent variable. In such cases, although the full mediation pathway is not established, the observed b-path can highlight the independent contribution of the mediator to the outcome and suggest the presence of confounding or parallel mechanisms that warrant further investigation.

A quantitative summary of all ten mediation models is provided in Table 2, showing standardized coefficients (*β*) and 95% confidence intervals for ACME, ADE, and total effects from bootstrap analysis (1000 iterations). Mediation paths are denoted as *X* → *M* → *Y* to clarify variable roles. Significant ACME values were observed for assocDyy, assocDzz, and projDyy, while most corpus callosum-mediated paths, especially those involving projDxx, were not significant. These results highlight the specific contribution of orthogonal diffusivity components—particularly within the ALPS-ROI—to the ALPS index.

**Table 2:** Standardized mediation effects for diffusion parameters on ALPS index

Path	ACME (*β*)	95% CI	ADE (*β*)	95% CI	Total effect (*β*)	95% CI
assocDxx → assocDyy → ALPS	652.88	[334.72, 1031.90]	−424.16	[−840.51, 26.30]	228.72	[−227.79, 743.80]
assocDxx → assocDzz → ALPS	−1045.76	[−1471.42, −656.60]	1274.48	[773.50, 1786.60]	228.72	[−248.79, 710.20]
projDxx → projDyy → ALPS	−751.31	[−1550.55, −210.90]	1293.70	[782.72, 1926.90]	542.40	[−260.32, 1207.20]
projDxx → projDzz → ALPS	−42.39	[−359.12, 144.30]	584.78	[−100.70, 1290.89]	542.40	[−197.01, 1191.49]
assocDxx → ccbDxx → ALPS	−19.03	[−100.64, 49.29]	247.75	[−236.51, 747.72]	228.72	[−249.13, 742.87]
assocDxx → ccbDyy → ALPS	−42.34	[−171.27, 105.63]	271.06	[−204.32, 745.57]	228.72	[−230.63, 710.72]
assocDxx → ccbDzz → ALPS	−30.69	[−195.83, 148.00]	259.41	[−243.16, 726.73]	228.72	[−267.47, 714.23]
projDxx → ccbDxx → ALPS	−87.50	[−315.02, 42.01]	629.90	[−120.11, 1235.03]	542.40	[−218.56, 1207.63]
projDxx → ccbDyy → ALPS	−24.97	[−275.76, 206.43]	567.36	[−152.89, 1193.44]	542.40	[−185.26, 1181.39]
projDxx → ccbDzz → ALPS	−108.52	[−405.12, 172.51]	650.91	[25.75, 1248.68]	542.40	[−167.01, 1244.44]

Table summarizes the standardized effects from mediation models using the format *X* (Independent Variable) → *M* (Mediator) → *Y* (Dependent Variable), where the outcome is the ALPS index. ACME (average causal mediation effect), ADE (average direct effect), and total effect are reported as standardized coefficients (*β*) with 95% confidence intervals (CI) computed via nonparametric bootstrapping (1000 simulations). Significant effects are indicated when the CI does not include zero

### Impact of the direction of medullary vessel on diffusivity

**Fig. 6:**
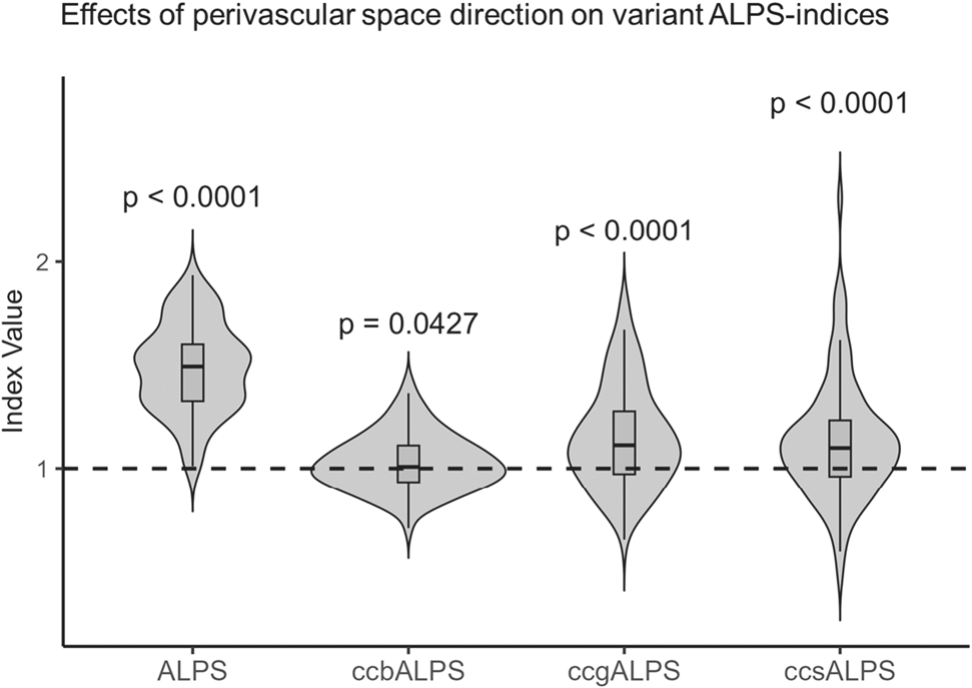
Variant ALPS indices in the corpus callosum. Results of analyzing the effect of perivascular space direction on the index by setting variant ALPS indices in the corpus callosum, which allows for a simpler evaluation of the relationship between white matter fibers and perivascular spaces. The values of the variant ALPS indices measured in the genu, body, and splenium of the corpus callosum were all significantly greater than 1, suggesting that the direction of the perivascular spaces affects diffusivity. Although the changes were smaller than the ALPS index in the ALPS-ROIs, these results indicate that the influence of perivascular spaces was present even within the corpus callosum

In an analysis that set the variant ALPS index in the corpus callosum, which was thought to allow for a simpler analysis of the relationship between white matter fibers and perivascular spaces of medullary vessels, and examined whether the direction of the perivascular spaces of medullary vessels affected the index (Fig. 6), although the changes were smaller than those in the ALPS index in the ALPS-ROIs, the values for the genu, body, and splenium were all significantly greater than 1. Thus, direction of the perivascular spaces of medullary vessels is thought to affect the ALPS index.

## Discussion

The MOONLIGHT study investigated the link between sleep and the glymphatic system using various imaging modalities. This report focuses on diffusion imaging parameters and volumetric measures derived from 3D T1-weighted images correlated with PSG-based sleep metrics. The ALPS index showed moderate inverse correlations with REM sleep latency and mild positive correlations with total sleep time and sleep efficiency. Among the variants, ccgALPS was inversely correlated with age and Stage N3, while corpus callosum body (ccb)-derived diffusivity measures showed positive associations with sleep efficiency. Furthermore, rCPV and rWMHV were positively correlated with age, sleep stage composition, and limb movement indices. Notably, ΔccsDzz was inversely correlated with Stage N3, and ΔrWMHV was positively associated with arousal and hypopnea indices. These findings suggest that both static and sleep-related changes in diffusion- and volume-based parameters may reflect alterations in brain microstructure and fluid dynamics during sleep. While correlation coefficients indicate effect size, p values (Fig. S1) reflect statistical reliability. Including both allows for balanced interpretation, as moderate correlations may not reach significance in studies with limited sample size.

The glymphatic system appears to be strongly influenced by physiological states such as sleep and anesthesia, which alter brain waste clearance. Studies in rats have shown that gadolinium-based contrast agents behave differently during sleep and wakefulness, indicating that sleep and anesthesia enhance brain clearance via the glymphatic system [40]. In older adults, poor sleep quality is correlated with a lower ALPS index, higher CPV, and cognitive decline, suggesting insufficient clearance mediated by the perivascular spaces [41]. Moreover, after the administration of the orexin receptor antagonist lemborexant, changes in the ALPS index and CPV are observed, along with improvements in sleep latency, total sleep time, and sleep efficiency [32]. In addition, amyloid imaging with ⁸F-Florbetaben reveals increased β-amyloid accumulation after sleep deprivation [42]. These findings imply that sleep disturbances could represent a “CNS-interstitial fluidopathy,” characterized by abnormal brain interstitial fluid dynamics [43, 44]. Conversely, a systematic review investigating how sleep quality and patterns affect glymphatic-related markers (including amyloid β, tau, α-synuclein, cerebrospinal fluid [CSF], PVS, and apolipoprotein E) in healthy adults reported inconsistent associations. Possible reasons for this include varied sleep assessment methods and reliance on self-reported data [45]. To clarify sleep–glymphatic links and ALPS index variations, integrated studies combining objective sleep evaluations and detailed analyses of brain water dynamics are needed.

Although this study linked the ALPS index to sleep, the ALPS method has been criticized. Concerns include a small ROI that may not represent the entire brain, instability in ROI placement causing measurement variability, and differences in imaging conditions [28, 35]. In addition, the ALPS-ROI may be influenced by commissural fibers oriented like perivascular spaces [26]. The assumption of radial symmetry has also been challenged: many brain areas show radial asymmetry (high λ2/λ3 ratio), likely due to crossing fibers [27, 46]. Finally, studies caution against interpreting the ALPS index as a direct glymphatic marker as it may be influenced by numerous other factors [25, 26, 28, 47].

We investigated the influence of white matter diffusion on the ALPS method, particularly focusing on commissural fibers that traverse the corpus callosum. Mediation analysis revealed that assocDyy and projDzz were associated with the ALPS index, suggesting that the index may reflect diffusion properties beyond those aligned with the perivascular space. The ccbDyy and ccbDzz values within the corpus callosum were also associated with the ALPS index, suggesting that the white matter microstructure involving commissural fibers may contribute to variability in the ALPS index. Therefore, the ALPS index should not be regarded as a specific marker of perivascular diffusivity, but rather as a metric influenced by multiple white matter structures. On the other hand, the variant ALPS indices measured in the genu, body, and splenium of the corpus callosum were all greater than 1.0, which may be consistent with the presence of diffusion along the perivascular spaces and indicating that diffusivity in the perivascular direction does contribute, at least in part, to the ALPS index. Taken together, these findings suggest that the ALPS index does not exclusively reflect perivascular diffusion, but is also dependent on the microstructural characteristics of widespread white matter fibers. Notably, abnormal ALPS indices have been reported in various disorders suspected of involving neurofluid dynamic abnormalities, supporting the notion that the ALPS index may have an indirect relationship with interstitial fluid flow [9–18]. Furthermore, it is worth noting that diffusion metrics other than the ALPS index also showed significant associations with both glymphatic markers and PSG-related parameters in the present study. Although previous large-scale morphometric studies have not identified consistent associations between sleep apnea and grey matter volume [48], the ALPS index may provide complementary insights by capturing diffusion-based microstructural features related to interstitial fluid dynamics. The observed correlations between the ALPS index and sleep parameters—along with its susceptibility to commissural fiber diffusivity—suggest that diffusion tensor imaging can reflect physiological alterations associated with sleep more sensitively than conventional volumetric approaches. These findings highlight the potential of DTI-based markers as valuable tools for assessing brain fluid dynamics.

It is suggested that the gaps or space between white matter fibers may act as drainage pathways for interstitial fluid [49]. Since DTI has become widespread, there has often been a misunderstanding that white matter fibers themselves are visualized, whereas in typical DTI (*b* = 1000), diffusion in the extracellular space is primarily hindered. DTI tracks water molecules constrained by tissue microstructure [50] and can be sensitive to astrocyte activity via aquaporin 4 [51]. Thus, diffusion imaging may indirectly indicate glymphatic activity [50]. Advection in the brain parenchyma is the fastest along privileged pathways, including the intraparenchymal perivascular spaces and white matter tracts, supporting large-molecule transport [49], suggesting a major role of white matter tracts in solute movement. Therefore, diffusion imaging inherently captures the aspects of glymphatic flow. The ALPS index likely produced favorable findings in previous studies because it gauges tissue diffusion in the deep white matter independent of specific brain functions. On the other side, although currently preferred, the ALPS approach is not the only way to evaluate glymphatic function using diffusion imaging [28]. Future studies should integrate multiple diffusion indices and carefully exclude regional- or function-specific influences.

This study has several limitations. First, it involved a unique psychiatric outpatient population with suspected OSA, limiting generalizability. Although we adjusted for psychiatric comorbidities using binary covariates, residual confounding from heterogeneous diagnoses and psychotropic medications cannot be ruled out. In addition, the study did not include an age-matched control group without OSA, limiting the ability to determine whether the observed diffusion alterations are specific to OSA or reflect general variability in the population. Second, the sample size was modest and from a single institution, restricting statistical power and preventing subgroup analyses. Third, although we applied the ALPS method using 12-direction single-shell DTI data, which has known limitations, this protocol has shown reproducibility in prior studies due to its focus on orthogonal fiber orientations. Fourth, only structural and diffusion MRI metrics were analyzed; other data collected in the MOONLIGHT study (e.g., biochemical and metabolic imaging) were not included and will be reported separately. Fifth, while ROIs were spatially normalized using non-linear registration, reliability metrics (e.g., Dice coefficients, ICC) were not computed. Sixth, the study included nine participants who underwent MRI after initiating CPAP, but CPAP effects were not explicitly modeled. Seventh, MRI was performed at two time points (16:00 and 09:00), which may have introduced unmodeled diurnal variation in diffusion and fluid metrics. Finally, due to its cross-sectional design, causal inferences cannot be made. Longitudinal studies with larger and more homogeneous samples are needed to validate our findings and further explore the effects of CPAP and psychotropic medications. In addition, portions of the manuscript were refined with the assistance of a generative language model (ChatGPT, OpenAI), under full authorial review and approval.

In conclusion, we investigated the relationships among the ALPS index, various diffusion parameters, and PSG-derived sleep metrics. The ALPS index demonstrated moderate correlations with REM latency, sleep efficiency, and total sleep time, but was also clearly influenced by diffusivity components unrelated to the perivascular direction, particularly in commissural fiber tracts. This suggests that the index reflects broader white matter microstructure rather than serving as a specific marker of perivascular diffusivity. Nonetheless, variant indices measured in the genu, body, and splenium of the corpus callosum all exceeded 1.0, indicating that diffusivity along perivascular spaces does contribute, at least in part, to the index. These findings underscore the dual nature of the ALPS index—being shaped by both perivascular orientation and the surrounding white matter architecture. Moreover, several non-ALPS diffusion metrics also showed significant associations with sleep-related and neurofluid markers, highlighting that white matter structure may play a role in interstitial fluid movement. While the ALPS index should not be interpreted as a highly specific marker of perivascular space function, it may still provide indirect insights into tissue-level fluid dynamics. A multimodal imaging approach that integrates regional diffusivity measures, volumetric indices, and physiological parameters is essential for a more accurate assessment of glymphatic function in the human brain.

## Supplementary Information

Below is the link to the electronic supplementary material.

Supplementary file1 (TIF 3592 KB)

Supplementary file2 (TIF 3518 KB)

Supplementary file3 (TIF 3296 KB)

Supplementary file4 (DOCX 13 KB)
