# Supplementary material for: Contribution of white matter microstructure to diffusion tensor image analysis along perivascular space in obstructive sleep apnea
Source: Jpn J Radiol. 2025 Jul 24;43(12):1926–41. doi: 10.1007/s11604-025-01838-x (PMC12647286; doi:10.1007/s11604-025-01838-x)
Supplement: Supplementary file 4 — Supplementary file4 (DOCX 13 KB) [file 11604_2025_1838_MOESM4_ESM.docx]

**Figure S1. P-value heatmap of correlations between imaging and PSG parameters**

The imaging parameters included the rCPV, rWMHV, ALPS indices (ccgALPS, ccbALPS, and ccsALPS), and diffusion indices for each region (ALPS-ROI and corpus callosum). The PSG parameters included sleep stages, respiratory indices, and sleep assessment indices. This heatmap presents the corresponding p-values from correlation analyses, visualized to indicate statistical reliability. Darker red shades represent lower p-values, with only values below 0.05 labeled; those below 0.001 are denoted as "< 0.001". While correlation coefficients reflect the strength of association, p-values indicate their statistical significance. Discrepancies may arise due to sample size or data variability and should be interpreted accordingly.

A. In MRI/4PM, significant associations (p < 0.001) were observed between rCPV or rWMHV and age, suggesting age-dependent enlargement of perivascular spaces and white matter hyperintensity burden. Additionally, the ALPS index showed a significant inverse association with REM sleep latency, and rCPV was associated with Stage N1 sleep.
B. In MRI/9AM, p-values indicated strong statistical associations between age and both rCPV and rWMHV, as well as several white matter diffusion indices. The ccsFA value was also significantly associated with Stage N3 sleep.
C. In Δ value analyses, although overall significance was limited, ΔrWMHV was significantly associated with age (p = 0.0005). Other Δ metrics, such as ΔassocDyy, showed moderate but statistically significant associations with REM sleep latency.
